# Epimax-Related Ocular Surface Toxicity (EROST): the Glasgow experience

**DOI:** 10.1038/s41433-023-02620-x

**Published:** 2023-07-06

**Authors:** Carl Mulholland, Elisabeth Macdonald, David Lockington

**Affiliations:** Tennent Institute of Ophthalmology, Gartnavel General Hospital, 1053 Great Western Road, Glasgow, G12 0YN UK

**Keywords:** Corneal diseases, Education

Atopic eczema is the most common chronic inflammatory skin condition (2–10% UK adult lifetime prevalence) [1]. Primary management involves frequent, liberal use of emollients and moisturisers to affected areas. In September 2021, NHS Greater Glasgow and Clyde’s formulary committee changed the first-line emollient management of atopic eczema to Epimax cream/ointment (Aspire Pharma). In early 2022 we became aware that an increasing number of dermatology patients were presenting to acute ophthalmological services with unexplained ocular surface toxicity, consistent with a mild ocular chemical injury. Further questioning identified symptoms were related to recent use of Epimax ointment/cream, similarly applied as previously used emollients. Our dermatology and pharmacy departments were unaware of similar issues with other emollients, and our literature search did not identify any published data. A retrospective case-note review of emergency eye clinic attendance involving such clinical presentations was undertaken to investigate this phenomenon.

**Fig. 1:**
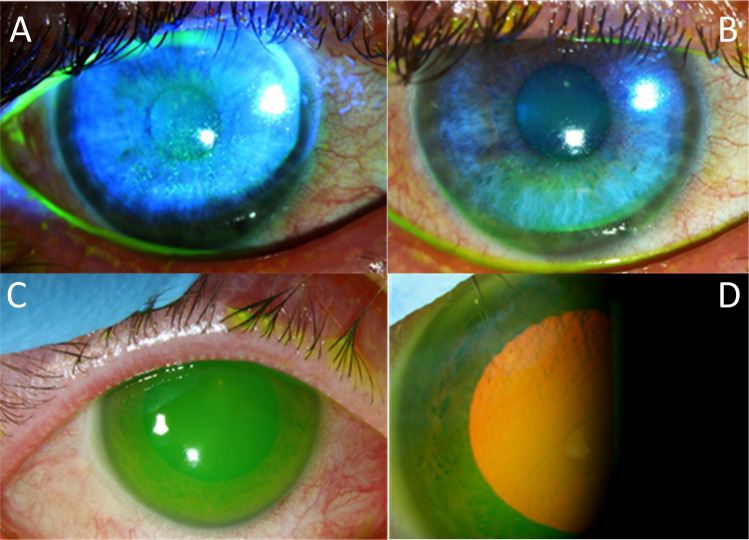
Composite figure of anterior segment photographs of Epimax related ocular surface toxicity. These presenting clinical features show corneal staining and haze (**A**, **B**), a total corneal defect (**C**) and corneal oedema and Descemet’s membrane folds (**D**).

We identified 37 patients with atopic eczema between January to October 2022 who attended with novel ocular surface toxicity, related in time-period (often <1week) to Epimax initiation (12 male:25 female; median age 42 years (range 8–95)). Most patients reported using Epimax ointment (23/37; 62.2%), with seven (18.9%) using Epimax original cream (seven unknown). Most reported subjectively reduced VA, frequently associated with photophobia (89.2%). Clinical findings included bilateral involvement (67.6%), with conjunctival injection (97.3%), corneal staining (97.3%), and corneal oedema (27%). Ocular pH was documented in seven initial cases (range 7–8). Four patients (10.8%) had complete/near complete corneal epithelial defects. No patients had limbal ischaemia. [See Fig. 1] One adult patient required overnight admission given the severity of bilateral ocular toxicity.

Following diagnosis, most patients received topical lubricants (86.5%), topical antibiotics (73%) and topical steroids (64.9%). Mean visual acuity improvement in affected eyes was 15.8 EDTRS letters with significant symptom resolution by first follow up appointment (average 7.3 days (range 2–34)). No geographic clustering was identified on postcode analysis, or association identified with Scottish Index of Multiple Deprivation scores (mean 2954.5; median 2417; range 33–6947).

**Fig. 2:**
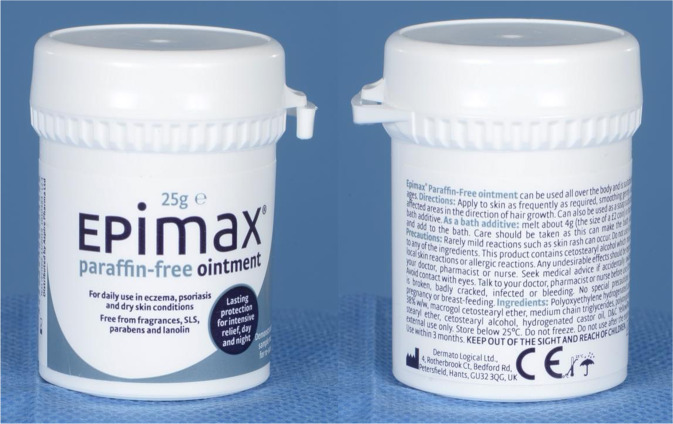
Photo of Epimax ointment. The packaging shows directions for use, list of ingredients and advice to “avoid contact with eyes”.

Like most emollients, the Epimax range contains safety information advising no contact with the eyes. [See Fig. 2] Aspire Pharma did not report any batch-specific or contamination issues [personal communication, October 2022]. Epimax ointment ingredients include liquid paraffin, yellow soft paraffin and emulsifying wax (cetostearyl alcohol and macrogol cetostearyl ether) [2]. Only cetostearyl alcohol has been reported to cause eye irritation [3]. 11.2% of eczema patients can have skin reactions to cetyl alcohol, and so could be more susceptible to ocular surface toxicity [4, 5]. Following discussion, an educational memorandum was issued to all local medical, nursing and pharmacy staff. We also reported our cases to the MHRA (despite Epimax technically classified as a medical device), and subsequently a Field Safety Notice reference 5032139 was issued on 20/01/2023 linking cetostearyl alcohol (a known skin sensitiser) to eye related toxicity [6].

In summary, we report the first large case-series of patients with eczema experiencing Epimax-related ocular surface toxicity (EROST). Patients should avoid periocular application of Epimax, and healthcare professionals made aware of this potential association.
